# The situation of elderly with cognitive impairment living at home during lockdown in the Corona-pandemic in Germany

**DOI:** 10.1186/s12877-020-01957-2

**Published:** 2020-12-29

**Authors:** Jochen René Thyrian, Friederike Kracht, Angela Nikelski, Melanie Boekholt, Fanny Schumacher-Schönert, Anika Rädke, Bernhard Michalowsky, Horst Christian Vollmar, Wolfgang Hoffmann, Francisca S. Rodriguez, Stefan H. Kreisel

**Affiliations:** 1 German Center for Neurodegenerative Diseases (DZNE), site Rostock/ Greifswald, Ellernholzstr. 1-2, 17489 Greifswald, Germany; 2 Institute for Community Medicine, Department of Epidemiology and Community Health, University Medicine Greifswald, Greifswald, Germany; 3 Division of Geriatric Psychiatry, Department of Psychiatry and Psychotherapy, Evangelisches Klinikum Bethel, University Hospital OWL – Campus Bielefeld-Bethel, Bielefeld, Germany; 4 Institute of General Practice and Family Medicine, Faculty of Medicine, Ruhr-University Bochum (RUB), Bochum, Germany

**Keywords:** COVID-19, Dementia, Germany, Social distancing

## Abstract

**Background:**

The outbreak of the Corona virus is a challenge for health care systems worldwide. The aim of this study is to analyze a) knowledge about, and feelings related to the Corona-pandemic. Describe b) loneliness, depression and anxiety and, c) the perceived, immediate impact of the lockdown on frequency of social contacts and quality of health care provision of people with cognitive impairment during social distancing and lockdown in the primary care system and living at home in Germany.

**Methods:**

This analysis is based on data of a telephone-based assessment in a convenience sample of *n* = 141 people with known cognitive impairment in the primary care setting. Data on e.g. cognitive and psychological status prior to the pandemic was available. Attitudes, knowledge about and perceived personal impact of the pandemic, social support, loneliness, anxiety, depression, change in the frequency of social activities due to the pandemic and perceived impact of the pandemic on health care related services were assessed during the time of lockdown.

**Results:**

The vast majority of participants are sufficiently informed about Corona (85%) and most think that the measures taken are appropriate (64%). A total of 11% shows one main symptom of a depression according to DSM-5. The frequency of depressive symptoms has not increased between the time before pandemic and lockdown in almost all participants. The sample shows minimal (65.0%) or low symptoms of anxiety (25%). The prevalence of loneliness is 10%. On average seven activities have decreased in frequency due to the pandemic. Social activities related to meeting people, dancing or visiting birthdays have decreased significantly. Talking with friends by phone and activities like gardening have increased. Utilization of health care services like day clinics, relief services and prescribed therapies have been reported to have worsened due to the pandemic. Visits to general practitioners decreased.

**Conclusions:**

The study shows a small impact of the pandemic on psychological variables like depression, anxiety and loneliness in the short-term in Germany. There is a decrease in social activities as expected. The impact on health care provision is prominent. There is a need for qualitative, in-depth studies to further interpret the results.

**Supplementary Information:**

The online version contains supplementary material available at 10.1186/s12877-020-01957-2.

## Background

The first cases of a novel atypical pneumonia – the infection later being named COVID-19 and shown to be caused by the corona virus strain SARS-CoV-2 – were reported in Wuhan, China in December 2019; the WHO announced it officially as pandemic on March 11, 2020, after the virus had rapidly spread to numerous countries worldwide. The first symptomatic case of a coronavirus SARS-CoV-2 infection in Germany was reported at the end of January 2020. The most common symptoms are dry cough, fever, fatigue, chest tightness, diarrhea, and dizziness [1] and the most severe symptoms include respiratory distress and septic shock. Reports had suggested that about 40% of the patients progress to severe illness within a time period of five days [2].

Scientific evidence has rapidly evolved during the last few months and is continuing to do so. However, it was clear from very early on that older adults and those with serious underlying medical conditions are at a higher risk for severe illness from COVID-19. Older people are more likely to present severe symptoms, fever, and breathing problems than younger people [3]. Comorbidity and lymphocyte count are the strongest predictors of progression to severity [2], with hypertension, diabetes, and higher body mass index as the most frequent comorbidities among patients with severe symptoms [1]. Accordingly, people who need extra precautions are, among others, elderly people with chronic diseases. A clinical review on testing, treatment and prognosis of COVID-19 in the elderly supports the association of age and presence of comorbidities with increased severity and mortality [4].

With no causal or symptomatic treatment being available, the aim of all protective measures has been to stop or slow down the pandemic and prevent people from becoming infected. This has been especially challenging as many of those infected remain asymptomatic or are infectious before symptoms appear. However, public health measures have been associated with the epidemiology of the disease [5]. General measures to encounter the pandemic were introduced in Germany starting March 22nd 2020 when the federal government in agreement with the governments of all federal states enacted limitations in social contacts (i.e. “social distancing”) throughout the country. Targeted social distancing is the standard practice to influence infection spread, especially for vulnerable groups because spread is strongest through social contact network [6]. While quarantine of infected people is a type of social distancing useful for many infectious diseases, social distancing on a broader community level aims at achieving minimal interaction between people in the population and is ethically more challenging [7]. Social distancing, also referred to as “physical distancing”, is described by the Center for Disease Control and Prevention (CDC) as “keeping space between yourself and other people outside of your home” https://www.cdc.gov/coronavirus/2019-ncov/prevent-getting-sick/social-distancing.html. The CDC recommends to a) stay at least a certain distance from other people, b) not gather in groups, and c) stay out of crowded places and avoid mass gatherings. Worldwide children were barred from going to schools; university classes, religious services, major events in sports, film industry, music festivals and concerts, conferences, and fashion shows were canceled [8]. In line with this definition, the German government recommended to reduce spatial closeness and decrease individual mobility as much as possible. In public places, a minimal distance between individuals of 1.50 m was introduced. Public gatherings of up to two people continued to be allowed (this rule, however, did not apply to people residing in the same household, e.g. families with more members could move freely in the public), public and private gatherings were forbidden, but people were generally “asked” to stay at home. In contrast to other countries, there was no strict, legally binding “lock down”. Furthermore, shops selling non-essential products, restaurants, clubs, schools, universities, public buildings, recreational facilities and other institutions of public life were closed. On April 15th some of the restrictions were eased (e.g. selected shops were allowed to open under specific restrictions), but social distancing was recommended to be kept in place until May 6th. Latter measure has now been extended until further notice. Due to the federal nature of state organization in Germany, recommendations and regulations were introduced jointly as a result of an inter-governmental agreement, but nonetheless posed only a non-binding framework for the federal states (the entities previously legally responsible for pandemic measures). Each federal state was able to develop specific plans, set different deadlines and interpret recommendations differently. Although the initial consensus was upheld in the early stages of the lockdown, federal diversity has increased lately. Starting May 6th 2020 it was mandated that the nation-wide approach in respect to preventive measures would be delegated to a more regional level and on 6th of June more restrictions were loosened. It has been shown that the provisions taken have contributed to the substantial reduction of the growth rate in Germany [9].

As the spread of COVID-19 is a specific challenge for the health care system, several measures have been introduced to deal with the pandemic in addition to those outlined above. The Robert-Koch-Institute, the German federal government’s central scientific institution for public health, has issued recommendations for hygiene for the public and the health care system in Germany. In this article, we focus on general recommendations that affect health care and might specifically affect the elderly. For example, to be prepared for an increased COVID-19 associated utilization of hospitals, the total number of beds for intensive care were increased, by e.g. postponing elective and planned hospital stays. Social distancing rules were applied to health care institutions and the primary care system. For example: day care clinics were closed, ambulatory service provision decreased, treatments cancelled or restricted in case these could not meet the recommended hygienic requirements (e.g. out-patient physiotherapy).

As of yet the consequences of these measures on medical, psychological or social variables during the pandemic remain unknown. Nonetheless, an impact can be expected. For instance, social distancing can be easily misunderstood as “distancing”; it is not meant to imply a reduction in social or emotional support [10]. Further, social distancing can be associated with a loss of motivation, loss of meaning, loss of self-worth, and subsequent mental health problems [11]. Moreover, the public dissemination of preventive measures can trigger anxieties, lead to discrimination and stigma, and cause misinformation through misunderstanding [12]. People with cognitive impairments might have difficulties to comprehend the imposed restrictions or may misinterpret the protective gear worn by health care professionals as threatening [13].

There is some scientific evidence from recent pandemics (SARS, H1N1, Ebola, MERS [14];) that highlight the impact of being “quarantined” on psychosocial factors. Among the negative outcomes were: depression, stress, irritability, sleep disorders, anger, etc. The highest prevalence is on irritability and depressive symptoms. In addition, relatives of people being quarantined experienced fear, nervousness, sadness and guilt. Albeit, there are also positive outcomes reported such as happiness and relief. Factors associated with negative outcomes were length of quarantine, frustration, boredom, fear of infection, undersupply of daily goods and information deficit.

A recent rapid literature review of the psychosocial impact of quarantine during pandemics implied extensive negative consequences for mental health [15]. Even though these results may be transportable to the impact of social distancing, there is a significant difference. Quarantine requires being infected or having had contact to a person infected whereas systematic social distancing is a measure with an impact at the population level.

Lack of social support, loneliness, and restricted social contact is associated with higher mortality [16] as well as a higher risk for cardiovascular disease [17] and dementia [18]. Moreover, among cancer patients, those with more social support had better survival chances [19]. This, what is considered to be a stress-buffering effect [20], is likely to be brought about by physiologic pathways related to neuroendocrine or immunologic function [21]. Social support appears to enhance the efficiency of the immune system in dealing with inflammatory processes [22, 23]. Furthermore, studies point out that neurons of socially isolated animals are less responsive to stimulation [24], emphasizing the relevance of social contacts for neurodegenerative processes. The health promoting effects of social contacts stand in contrast to the vulnerability of older people to be lonely [25]. There is outspoken concern about the effect of isolating the elderly given the pandemic and beyond [26] and the German Association of Gerontology and Geriatrics has published a statement to maintain participation and social inclusion for the elderly during the Corona-pandemic [27].

A description and analysis of the perceived impact of the pandemic on the population at risk is needed to add scientific evidence and in the end support (future) healthcare to provide appropriate measures [28]. Therefore, the aim of this study is to:

- Analyze knowledge about, and feelings related to the Corona-pandemic.
- Analyze loneliness, depression and anxiety of people with cognitive impairment during lockdown.
- Analyze the perceived, immediate impact of the lockdown on frequency of social contacts and quality of health care provision in a cohort of people with cognitive impairment in the primary care system and living at home in Germany.

## Methods

This analysis is based on data of a telephone-based questionnaire and semi-structured interview assessment in a convenience sample of *n* = 141 people with cognitive impairment in the primary care setting in Mecklenburg-Western Pomerania and North-Rhine-Westphalia. Participants were recruited from currently running interventional trials intersec-CM (Supporting elderly people with cognitive impairment during and after hospital stays with intersectoral care management) [29] and the DCM:IMPact study (Dementia Care Management: Implementation into different Care Settings), an implementation study of the dementia care management of the DelpHi-MV trial [30–32]. Ethical approval has been obtained from the ethics committee of Greifswald Medical School (registry number: BB 159/17) and the ethics committee of the Chamber of Physicians of Westfalen-Lippe (registry number: 2017–688-b-S). Ethical approval for the DCM:Impact study was obtained by the ethics committee of the University Medicine Greifswald (BB 01/2019). Participants gave written informed consent to participate in these studies. In both studies, participants agreed to be regularly asked about their current living situation, health, quality of life, social contacts, health issues, burden, utilization of medical services and others. As such, conducting the assessments analyzed here were covered by the original ethical approval and no further approval was obtained. Written informed consent was obtained personally or by legal guardians when necessary. The assessments of the present analysis were conducted by trained study staff during the time of the lockdown during study contacts between 22nd of March and 5th of June 2020, before restrictions were lifted.

### Sample

A total of *n* = 115 participants of the intersec-CM trial received the additional assessment. Intersec-CM is a complex, longitudinal, multisite randomized controlled trial. It was designed to treat a hospital-based cohort of people above the age of 70 with an adaption of “Dementia Care Management” (DCM) – a treatment proven to be effective in primary care – to the discharge setting. As part of this, specifically trained study staff develops, implements, and monitors a treatment and care plan, based on comprehensive assessments during the hospital stay, recommendations at discharge and unmet needs at home. For the 3 months after discharge study staff coordinates treatment and care in close cooperation with the discharging hospital, treating physician and other care providers. The first patient from a total of *n* = 401 was recruited in November 2018, the last in April 2020. Participants of the ongoing intersec-CM trial were asked to take part in the additional assessment only if they belonged to the original study’s intervention group or were part of the control group after completion of their individual follow-up time period.

A total of *n* = 26 participants were interviewed in the framework of the DCM:IMPact study, a mixed-methods, multi-center, implementation study. DCM:IMPact compliments the DelpHi-trial (Dementia: Life- and person-centered help). In this study, the effective [31] and cost-effective [32] dementia care management is implemented in different care settings, including ambulatory care services, dementia care networks, and hospitals, aiming to reveal in which care setting the highest need and lowest implementation barriers for such model of care exist, and thus the highest effects could be achieved. The DCM:IMPact study started in 2019.

Owing to this particular ascertainment scheme (the localities in which the above studies were performed), participants come from two German federal states: Nordrhein-Westfalen and Mecklenburg-Vorpommern.

### Data assessment

The benefit of interviewing a known convenience sample is that baseline sociodemographic characteristics and certain clinical variables are available. Variables that usually cannot be assessed by phone, like cognitive status, are known. The sample is described in detail in Table 1. Additionally, study participants have an established relationship with the interviewers. We expect this personal contact to increase the validity of the data obtained. In case of uncertainty about the validity of the data provided by the person with cognitive impairment, the caregiver was asked to provide information..

**Table 1:** Sample (*n* = 141)

			Male (51)		Female (82)		Total	
Variable	Value	range	n/m	%/SD	n/m	%/SD	n/m	%/SD
Sex	Female						82	61.7%
Age	Years	64–98	80.16	6.67	82.34	6.09	81.52	6.38
Living situation	Living alone (yes)		23	45.1%	22	27.2%	46	34.1%
Cognitive status	MMSE-score (0–30)	7–29	23.02	3.11	23.17	3.75	23.12	3.52
Instrumental social support	Total (0–12)	2–12	9.29	2.47	9.82	2.14	9.60	2.29
	Support in repairing something (0–3)	0–3	2.0	0.89	2.22	0.87	2.15	.88
	Shopping support (0–3)	1–3	2.64	0.63	2.58	0.67	2.60	0.65
	Support in filling out forms (0–3)	0–3	2.26	0.63	2.31	0.81	2.29	0.80
	Support in dealing with administration (0–3)	0–3	2.28	0.78	2.37	0.75	2.33	0.76
Loneliness	Yes							
	De Jong Gierveld-score (6–24)	6–20	10.73	3.64	11.35	3.06	11.12	3.24
	Hughes-score (3–9)	3–9	4.34	1.71	4.62	1.96	4.45	1.80
	Considered lonely (DZA)		4	10.3%	7	10.3%	11	10.2%
Anxiety symptoms	GAD-7 score (0–20)	0–18	3.64	3.67	3.79	2.95	3.73	3.22
	Minimal symptoms		34	72.3%	50	62.5%	84	65.1%
	Low symptoms		8	17.0%	24	30.0%	32	25.2%
	Medium symptoms		5	10.6%	6	7.5%	11	8.7%
	Severe symptoms							
Anxiety disorder	Possible (> 10)		5	9.8%	2	2.4%	7	5.3%
	Probable (> 15)		0	0	0	0	00	0
One main symptom of depression according to DSM-5	Yes		5	9.8%	10	12.20%	15	11.37%
Sadness (*n* = 135)	Never		33	70.2%	43	53.8%	76	59.8%
	Single days		13	27.7%	33	41.3%	46	36.2%
	More than half the days		1	2.1%	3	3.8%	4	3.1%
	nearly daily		0	0	1	1.3%	1	0.8%
Loss of interest (n = 135)	Never		22	47.8%	43	53.8%	65	51.6%
	Single days		19	41.3%	28	35.0%	47	37.3%
	More than half the days		5	10.9%	7	8.8%	12	9.5%
	nearly daily		0	0%	2	2.5%	2	1.6%
Change in sadness (*n* = 102)	Sadness more often		0	0%	0	0%	0	0%
	Sadness equally often		0	0%	2	3.0%	2	2.0%
	Sadness less often		35	100.0%	65	97.0%	100	98.0%
Change in loss of interest (n = 102)	Loss of interest more often		0	0%	1	1.5%	1	1.0%
	Loss of interest equally often		1	2.9%	5	7.5%	6	5.9%
	Loss of interest less often		34	97.1%	61	91.0%	95	93.1%
(Social) activities (24)	With a perceived increase in frequency	0–7	1.07	1.04	1.37	1.38	1.26	1.27
	With a perceived decrease in frequency	0–16	6.12	3.92	7.27	3.79	6.83	3.97
	With no perceived change	0–15	6.67	3.60	5.27	2.89	5.80	3.24
	n/a	0–24	9.84	4.74	10.4	4.57	9.96	4.56
Healthcare-related services (11)	With a perceived negative impact	0–5	0.86	1.02	1.12	1.27	1.02	1.18
	With no perceived change	0–11	3.75	2.50	4.02	2.24	3.92	2.34
	With a perceived positive impact	0–2	0.20	0.49	0.18	0.45	0.19	0.46
	n/a	0–10	6.18	2.35	5.59	2.31	5.81	2.34

*Footnote: n number of participants, m mean, SD standard deviation, n/a not applicable*

From end of March until 6th of June, we conducted a telephone-based questionnaire and semi-structured interview assessment. The questionnaire included variables from previously validated instruments and is available as supplement (supplement 1). They were chosen based on their feasibility in telephone-based surveys, duration and feasibility in people with cognitive impairments. All were available in German versions and conducted in German. The decision for inclusion was based on an expert panel of researchers at the DZNE, the university of Greifswald, the university of Rostock, the university of Bochum and experts from the geriatric division of the hospital in Bielefeld. They cover:

- Attitudes, knowledge about and perceived personal impact of the pandemic
- social support
- loneliness
- anxiety
- depression
- change in the frequency of social activities due to the pandemic
- perceived impact of the pandemic on health care related services
  Ad a) Participants were asked whether they were sufficiently informed about Corona (yes/ no) and whether they knew someone infected (yes/ no) and whether they rated the measures against the pandemic as appropriate (“Yes”, “No – too strict”, “No – not strict enough”). They were asked to rate whether they felt “1 – strongly”, “2 – moderately”, “3 – not at all” worried because of Corona for themselves; “stressed out”, “concerned about their health”, “the health of family and friends”, “worried about Corona in general”. They were asked to rate their fear to infect family members, whether their everyday life has changed by the pandemic and whether the visiting restrictions for nursing homes do affect their life. The items are listed in Table 2.
  Ad b) Social support was measured using selected items from the German version of the Resource Generator [33]. The Resource Generator covers a wide variety of social resources that can be divided in four aspects of social capital (capital associated to: prestige and education of the network, political and financial knowledge of the network, special skills of the network, social support of the network). For our analysis and due to time restrictions in the assessment four relevant items from the aspect “instrumental social support” were chosen (i.e. is there anyone, who “supports you when repairing something”, “supports you when shopping”, “supports you in filling out forms”, “supports you with legal and administrative challenges”). The participant was asked to rate these with: “0 – not at all”, “1 – rather not”, “2 – rather”, “3 – exactly”. Each item is analyzed separately and for the purpose of analysis, we add the scores of each item to generate a total score of social support, ranging from 0 to 12.
  Ad c) Loneliness was measured using the loneliness scale of de Jong Gierveld & van Tilburg [34]. This scale consists of 6 items with statements the participant is asked to rate with “1 - exactly”, “2 - rather”, “3 - rather not” or “4 - not at all”. The scores of each item are summed to a total score, ranging from 6 to 24; the higher the score the higher loneliness is present in the participant. This instrument was chosen, because it is used in the longitudinal population-based cohort of the elderly in Germany and reference scores are available for the years 2008 to 2017 [35]. We furthermore measured loneliness with a focus on social relationship using a short loneliness scale developed specifically for use on a telephone survey [36]. Three items ask how often one feels “lacking companionship”, “being left out” and “being isolated”. Each item is rated “seldom”, “sometimes” or “often”. The sum score indicates the level of loneliness, ranging from 3 to 9.
  Ad d) Anxiety is measured using the Generalized Anxiety Disorder Scale-7 (GAD-7) [37]. It is a screening instrument to diagnose a generalized anxiety disorder according to DSM-5 and ICD 10. The psychometric properties are considered appropriate. The German version is validated for the general population [38]. Participants are asked to rate how frequent symptoms of anxiety have occurred during the time-frame of 2 weeks preceding the interview (“0 – not at all”, “1 – single days”, “2 – more than half of the days”, “3 – nearly daily”). The GAD-7 delivers a sum score by adding each item and ranges from 0 to 21. The sum score yields whether anxiety symptoms are minimal (0–4), low (5–9), medium (10–14) or severe (15–21). Furthermore, there are cut-off scores for a generalized anxiety disorder being possible (score > 10) or probable (score > 15).
  Ad e) The two item Patient Health Questionnaire (PHQ-2) was used for screening for depressive symptoms [39, 40]. The participant is asked to categorize how often he/ she has experienced “loss of interest or pleasure in activities” and “sadness” during the 2 weeks prior to the assessment. The options are “0 – never”, “1 – single days”, “2 – more than half of the days”, 3 – nearly daily”. These two items represent the main indicators for a positive screening of depression according to DSM-5, with one of both being mandatory. Depression was considered possible if one of the two items was scored > = 2. Depression was considered unlikely otherwise. For the sample of participants recruited for the intersec-CM study we included pre-pandemic data and could therefore calculate whether there was a change in frequency of symptoms during the period of lockdown (“more frequent”, “equally frequent”, “less frequent”).
  Ad f) To measure change in frequency of (social) activities due to the pandemic we use a generic list of social activities and ask whether the specific activity is carried out “more frequently”, “less frequently” or “with no change in frequency” during the time of lockdown. If an item does not apply to the participant’s situation, it was marked as being “not applicable” (e.g. if a participant never “went dancing” previously, this item was marked as “not-applicable”). The list consists of 24 items (see Table 3). Each item is analyzed separately. We furthermore provide three scores per person, which represent the number of activities that have “increased”, “decreased”, and been “unchanged” in frequency.
  Ad g) To assess the perceived impact of the pandemic on health care related services participants are asked to rate whether the provision and use of the service is “better”, “worse” or “unchanged” during the lockdown in comparison to the time before. Items that were irrelevant to a participant’s situation were marked “not applicable” (e.g. if a participant never utilized “podiatry” as a service, this item was marked as “not applicable”). The full list is provided in Table 4. Each service is analyzed separately. We furthermore provide three scores per person, which represent the number of services where the quality is rated to be “better”, “worse” and “unchanged”.

**Table 2:** Attitudes, knowledge about and perceived personal impact of the pandemic in a sample of n = 141 elderly with prior cognitive impairment

Statement	response	n	%
Are you sufficiently informed about Corona?	Yes	119	85.0%
Do you know anyone infected with Corona?	Yes	4	2.9%%
Do you think the measures against the pandemic are appropriate? (n = 135)	Yes	90	64.3%
	No, too strict	15	10.7%
	No, not strict enough	17	12.1%%
Do you feel worried for yourself because of Corona?	Strongly	16	11.4%
	Moderate	74	52.9%
	Not at all	50	35.7%
How stressed out do you feel because of Corona?	Strongly	6	4.3%
	Moderate	44	31.7%
	Not at all	89	64.0%
How concerned are you about your own health because of Corona?	Strongly	16	11.4%
	Moderate	62	44.3%
	Not at all	62	44.3%
How concerned are you about your family’s and friend’s health because of Corona?	Strongly	21	15.1%
	Moderate	76	54.7%
	Not at all	42	30.2%
How much do you fear about carrying the infection to a relative?	Strongly	7	5.0%
	Moderate	24	17.3%
	Not at all	108	77.7%
Do you worry about Corona in general?	Strongly	10	7.2%
	Moderate	69	49.6%
	Not at all	60	43.2%
Has your everyday life changed by the pandemic?	Strongly	15	10.7%
	Moderate	44	31.4%
	Not at all	79	56.4%
Have the visiting restrictions for nursing homes affect your life?	Strongly	13	9.3%
	Moderate	7	5.0%
	Not at all	120	85.7%

**Table 3:** Perceived change in frequency of social activities during the lockdown in Germany by a sample of n = 141 elderly with prior cognitive impairment

(Social) activity	More frequently		Less frequently		Unchanged		Not applicable	
	n	%	n	%	n	%	n	%
Being visited by family	9	6.4%	65	46.4%	49	35.0%	17	12.1%
Being visited by neighbors	0	0%	73	52.1%	29	20.7%	38	27.1%
Being visited by friend	3	2.1%	84	60.0%	20	14.3%	33	23.6%
Meet relatives	0	0%	86	61.4%	18	12.9%	36	25.7%
Meet friend	0	0%	86	61.4%	13	9.3%	41	29.3%
Visit birthdays/ festivities	1	0.7%	88	62.9%	11	7.9%	40	28.6%
Visit hairdresser	0	0%	87	62.1%	15	10.7%	38	27.1%
Make music	1	0.7%	9	6.4%	7	5.0%	123	87.9%
Go dancing	0	0%	11	7.9%	0	0%	129	92.1%
Go for coffee	1	0.7%	81	57.9%	5	3.6%	53	37.9%
Spend time outside	20	14.3%	49	35.0%	61	43.6%	10	7.1%
Go shopping	2	1.4%	69	49.3%	35	25.0%	34	24.3%
Talk with friends and relatives by phone	53	37.9%	1	0.7%	83	59.3%	3	2.1%
Watch TV	31	22.1%	10	7.1%	98	70.0%	1	0.7%
Clean the house	8	5.7%	8	5.7%	98	70.0%	1	0.7%
Work in the garden	13	9.2%	5	3.5%	31	22.0%	92	65.2%
Home improvement	5	3.5%	3	2.1%	22	15.6%	111	78.7%
Knitting, other crafts	7	5.0%	1	0.7%	28	19.9%	105	74.5%
Visit the GP	3	2.1%	46	32.9%	58	41.1%	33	23.6%
Visted by the GP	3	2.1%	17	12.1%	21	14.9%	100	70.9%
Visit medical specialist	4	2.8%	41	29.1%	64	45.4%	32	22.7%
Visit public institutions	0	0%	19	13.5%	32	22.7%	90	63.8%
Use computer/ tablet	7	5.0%	1	0.7%	30	21.3%	103	73.0%
Utilize voluntary services	1	0.7%	6	4.4%	4	2.9%	125	91.9%

**Table 4:** Perceived impact of the lockdown on health care provision by a sample of n = 141 elderly people in Germany

Health care services	Worse		Unchanged		Better		Not applicable	
	n	%	n	%	n	%	n	%
Ambulatory care service	8	5.7%	54	38.3%	5	3.5%	74	52.2%
Physiotherapy	10	7.1%	32	22.7%	1	0.7%	98	69.5%
Podiatry	30	21.3%	48	34.0%	0	0%	63	44.7%
Prescribed therapies	20	14.2%	36	25.5%	2	1.4%	82	58.2%
Hospital treatments	9	6.4%	44	31.2%	2	1.4%	84	59.6%
Day clinics/ services	8	5.7%	4	2.8%	1	0.7%	127	90.1%
Provision of medication	3	2.1%	129	91.5%	4	2.8%	4	2.8%
Utilities	6	4.3%	68	48.2%	2	1.4%	64	45.4%
Food service	1	0.7%	21	14.9%	1	0.7%	11	83.0%
Mobility	28	19.9%	92	65.2%	1	0.7%	20	14.2%
Relief services	16	11.3%	19	13.5%	6	4.3%	99	70.2%

## Statistical analysis

In this exploratory study we provide descriptive statistics for the variables under analysis. Descriptive analyses were conducted using IBM SPSS Statistics 21. Participants with missing data in single items were excluded from the analysis of this special item or the scale this item is part of. No data were imputed.

## Results

### Sociodemographic and clinical characteristics

The final sample is on average 81.5 years old and consists of 61.7% women. It includes mainly people with mild dementia (mean MMSE score of 23.1). Around one third of the sample lives alone (34.1%). Women, on average, were two years older and were less likely to live alone (27.2% vs 45.1% in males).

### Attitudes, knowledge and personal impact of the pandemic

The vast majority of participants report that they are sufficiently informed about Corona (83.5%) and most think that the measures taken are appropriate (62.1%). Nonetheless, 26.2% think they are inappropriate, but this group divides half into “too strict” and half into “not strict enough”. Most people do generally not worry (48.5%) or worry moderately (42.7%) about Corona. In respect to their own risk, most people feel moderately worried (51.5%) or not worried at all (36.9%). Approximately the same distribution is found for specific worries about own health, family and friends, fear for infection, fear of infecting others, and change of everyday life. The detailed results are shown in Table 2.

### Loneliness, anxiety, and depression

Analyzing measures of psychological status (i.e. loneliness, anxiety, and depression), the cohort shows sadness “never” in more than half of the people (59.8%) or on “single days” (36.2%). There is also “never” loss of interest in 51.6% of the sample. A total of *n* = 15 (11.4%) show one main symptom of depression according to DSM-5. The cohort shows minimal (65.1%) or low (25.2%) symptoms of anxiety..The mean sum score on the GAD-7 is 3.2 (range: 0–18; SD = 3.2). Possible anxiety disorder (GAD-7 score > 10) is found in *n* = 7 participants. It is more frequently observed in males (9.8%) than in females (2.4%). According to the loneliness-score of De Jong Gierveld 10.2% of our cohort are considered lonely, the mean loneliness scores being 11.1 (range 6–20, SD = 3.2); participants scored 4.5 on the Hughes scale (range 3–9, SD = 1.8). See Table 1 for detailed results.

### Social support and social activities

**Fig. 1:**
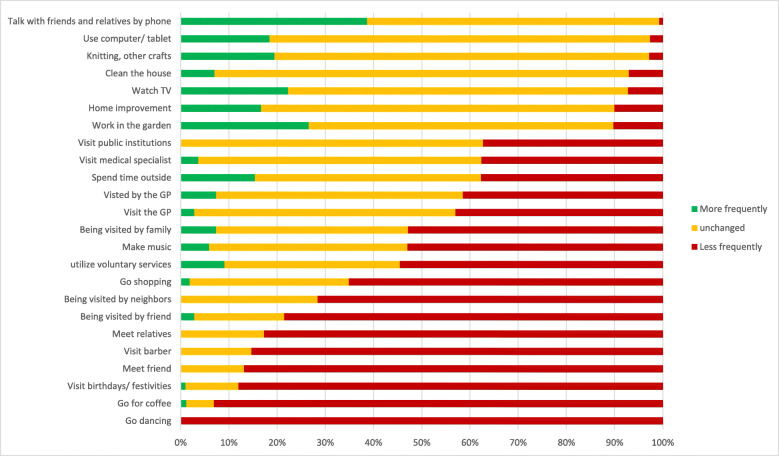
Change in frequency of (social) activities during the Corona pandemic in Germany in a sample of n = 141 elderly with prior cognitive impairment Footnote: the bars represent the distribution of answers by participants that showed the activity before the pandemic. Participants that indicated “not applicable” were excluded

Instrumental social support in this sample is high. The scale ranges from 0 to 3 per dimension and the mean is higher than 2 in all social support dimensions measured. The total sum of social support shows a mean of 9.6 (range 2–12, SD = 2.3). The interviewed reported on average 1.3 distinct social activities with an increase in their pursuit during the time of pandemic (SD = 1.3), on average 6.8 activities were pursued with a decreased frequency (SD = 4.0) and on average 5.1 (SD = 3.2) were carried out with the same frequency as before. On average *n* = 10.0 activities were indicated to not be relevant to the individual participant. Among the activities that have increased in frequency are: “Talk with friends and relatives by phone” (37.9% of the total sample), “Watch TV” (22.1%), “Spend time outside” (14.3%), “Work in the garden” (9.2%), “Being visited by family” (6.4%), “Knitting” (5.0%) and “Using computer/ tablet” (5.0%). Activities that decreased in frequency are: “Visit birthdays/ festivities” (62.9%), “Visit hairdresser” (62.1%), “Meet friends” (61.4%), “Meet relatives” (61.4%), “Go for coffee” (57.9%), and “Being visited by neighbors” (52.1%). More details are found in Table 3 and illustrated in Fig. 1.

**Fig. 2:**
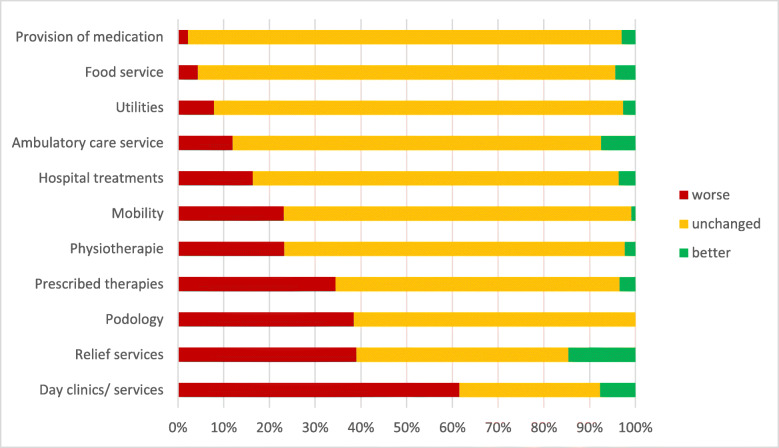
Perceived impact of the lockdown on health care provision by a sample of *n* = 141 elderly with prior cognitive impairment. Footnote: the bars represent the distribution of answers by participants that showed the activity before the pandemic. Participants that indicated “not applicable” were excluded

If one limits the analysis only to those activities that were reported to be relevant to each participant (i.e. removing individual “non-applicables”) the results are remarkably different. This is visualized in Fig. 2. Every participant indicated that “Go dancing” had decreased due to the Corona pandemic. More than 70% indicated that “Going for coffee”, “Visiting birthdays/ festivities” or “Visiting the hairdresser”, “Meeting friends and relatives” and “Visits by friends and neighbors” had decreased due to Corona. On the other hand, the frequency of “Talking with friends and relatives by phone” increased in approximately 40% of the participants and did not decrease relevantly in the others.

### Impact on health care related services

Services that were perceived as being worse were: “Podiatry” (21.3%), “Prescribed therapies” (14.2%) and “Relief services” (11.3%). Almost no health-care-related items were perceived as having improved during the pandemic (m = 0.2, range 0–2, SD = 0.5). Those that were nonetheless reported to be used/available more often were: “Relief services” (4.3%), “Ambulatory care services” (3.5%), and “Provision of medication” (2.8%). See Table 4 for more details.

Again limiting the analysis only to relevant health-related services (i.e. removing individual “non-applicables”), the results change somewhat (see Fig. 2). Services described as having been utilized less often during the lockdown by more than 30% were “Day clinics”, “Relief services”, utilization of “Prescribed therapies” and “Podiatry”. There is a small proportion of participants indicating that “Ambulatory care services”, “Day clinics” and “Relief services” had improved.

## Discussion

Our results reflect the situation of elderly in primary care, living at home during the lockdown imposed on them by the Corona pandemic in Germany.

### Knowledge about, and feelings related to the Corona-pandemic

The evidence shows that most elderly perceive their knowledge about the virus as being sufficient, indicating that the public information policy was adequate. That is important as research has shown that lack of knowledge in quarantine is a risk factor for later negative health outcomes [14]. In general, the cohort is modestly worried to not worried about themselves, their health or their relatives. Of note: None of those interviewed were infected themselves, only few knew others that had been infected. This fact might partly explain the low level of reported worries. Other explanations are also possible and may reflect aspects of age and life experience. Notes from semi-structured interviews accompanying the questionnaire can be interpreted in this light. Participants commented the situation as follows: “I am not afraid. I am 93, you can only die once! [Ich habe keine Angst, bin jetzt 93, einmal kann man nur sterben]” or “We have already lived through much worse situations [Wir haben schon Schlimmeres erlebt]“.

Nonetheless, agreement with the measures taken by the government was very high. It is unclear what drives this consensus. On the one hand, the active and transparent health communication adopted by policy makers might have contributed. On the other hand, generation-specific experiences or age-associated attitudes may have been beneficial. One participant explained it saying: “Queen Elizabeth has given courage and hope in her address [Queen Elizabeth hat in ihrer Ansprache Mut und Hoffnung gemacht]”, reflecting the, in the German media, well-publicized speech by Queen Elizabeth II on the April 5th.

### Loneliness, depression and anxiety of people with cognitive impairment during lockdown

Participants of our study show levels of loneliness, anxiety and depression that are comparable to people in the general population – during “normal” times. In Germany, the prevalence of loneliness in the general population of 45–84 year olds is 9.2%, in the age group presented here it is between 7.5 and 8.1% [35]. This is comparable to the 10.2% we found in our study. Loneliness being a rather stable construct may explain this result. A short-term isolation or restriction of social contacts with a clear cause and a foreseeable end might not influence the feeling of being lonely. It might have a differential effect on lonely people. .

Remarkably, depression and anxiety scores were lower than would be expected in the general population – outside of the pandemic. The prevalence of any anxiety disorder according to DSM-IV has been estimated to be around 10% in the general population [41]. Our results are much lower. The same holds for depression. The German population-based Leila75+ study showed a prevalence of depression of 38.2% in the 75+ year olds [42] and, the LIFE study with participants of the age from 18 to 80 a prevalence of depression of 6.4% [43]. In our cohort, we assessed prevalence (11.4%) using a cutoff of 2 or more points on one item of the PHQ-2; this potentially even overestimates the true prevalence of depression in our cohort.

However, these results need interpretation. Prevalence data for Germany is population-based. Our study, on the other hand, is based on a convenience sample of people already participating in intervention trials. As such, a selection bias is possible in that people with a previous diagnosis of depression or anxiety disorders might not have participated in the original study. This notwithstanding, approximately half of the cohort showed single depressive symptoms at baseline. Comparing these to the pandemic results of the two PHQ-2 items “loss of interest or pleasure in activities” and “sadness” (in intersec-CM participants), our results imply that the pandemic did not impact symptoms of depression. In fact, descriptively depressive symptoms before the baseline were shown to be more frequent than during the lockdown. Again, this may not reflect a shift causal as all intersec-CM participants were initially recruited during an acute hospital stay. Summarizing, we can show that depressive symptoms are present in our cohort, but there do not seem to be caused by the Corona pandemic and the restrictions imposed.

### Impact of the lockdown on frequency of social contacts, social activities and quality of health care provision

The level of social isolation in the general population of elderly in Germany has been reported to be around 13%, in the age group of 60+ up to 20% [44]. Due to limitations of the telephone interview setting, we were only able to assess instrumental social support as a dimension of social isolation. On average, our cohort received medium to high levels of instrumental support even under lockdown-conditions and therefore cannot be considered isolated. We asked whether the frequency of pursuit in diverse social activities had changed due to the pandemic. On average the frequency did not change in six activities, one was pursued more and seven less often. We can conclude that the level of social activities in the elderly in our cohort did not change significantly; restrictions in activities that are important to a given individual (especially those related to “staying in contact”) were, in fact, compensated during the lockdown.

Activities pursued less frequently are associated with activities that are difficult to perform given social distancing. Participants met with other people less frequently, went out less frequently and visited social gatherings less frequently. However, the pandemic had a “positive effect” by increasing the frequency of talking to friends and relatives by phone, gardening or similar activities with less personal contact. Thus, the elderly find various ways to maintain social relations and stay in contact with their relatives and friends. Due to this, pandemic-related restrictions might not have direct and short-term consequences on measures of loneliness and social isolation. The elderly studied here – notable that they all suffered from impaired cognition (as a result of the population the cohort was recruited from) – seem to retain sufficient resources to keep themselves occupied, irrespective of the pandemic.

The strongest impact of the restrictions imposed on the elderly in our cohort were the ones referring to the provision and utilization of health care services. Our participants visited or were visited less by general physicians or other medical specialists. They rated the provision of ambulatory services, day clinics and prescribed therapies as worse due to the pandemic. Especially the provision of services aiming at relief for caregivers was perceived to be worse. These results need further attention as a decrease in health care services may lead to significant long-term consequences. Recent findings from a study in the UK highlight the need to encounter this challenge. They found that paid home care was significantly affected by family members’ decisions on whether care should be continued or not [45]. The authors recommend that the health care system needs to be prepared for this by improving guidance and logistical support.

We do not have any systematic information about the specific reasons participants rated the change in activity pursuit the way they did. From the semi-structured interviews, however, there is some indication that causes are inter-individually quite diverse. For example, one participant explained that his prescription for a sleep apnea device was stopped “due to” Corona. Others reported that ambulatory care services reduced their services of fear of infecting their clients or of becoming infected themselves. This had substantial consequences especially in those with mobility restrictions needing help when shopping or simply aid when walking. A more in-depth qualitative assessment is needed here.

Elderly people, who do not utilize health care services for a certain period of time endanger their health and the health care system might have high treatment costs. This might be devastating for the German health care system. A decreased number of patients admitted to the hospital, GPs or specialists within the Corona-pandemic leads to lower income for health care providers and, in the short-term to lower health care expenditures. However, as a result of the non-use of health care services, there will probably be significantly more serious cases in the clinics in the future and more patients with severe pre-existing conditions. This increases long-term treatment costs and can put enormous strain on the health care system. Only in a few years, the economic effects and consequences of the pandemic (i.e. high treatment costs and life-threatening diagnoses) can be recorded exactly.

Besides that, more attention must be focused on the needs of the caregivers. It is well documented that caregiver burden is high and a risk factor for institutionalization [46–48]. Services aiming at relief for caregivers have been established to support caregivers and ease the situation for people living at home. With hindering and/ or closing these services the burden on caregivers will have increased. This is especially the case when they now compensate for professional services that are restricted or no longer provided given the pandemic. Thus, it is possible that caregiver health outcomes will be detrimentally effected. There is an urgent need to look more closely and monitor this potential development.

### Limitations

There are clear limitations to the results presented in this study and restrict their generalizability.

The impact of the pandemic on institutionalized elderly has been reported to be severe, such as high rates of positively tested residents of nursing homes, higher mortality [49]. Isolating care home residents in their rooms is associated with morbidity and raises patient safety and staffing issues [50]. The SARS-CoV-2 pandemic could also have lasting psychological impacts on care home staff [50]. It is important that support is provided. Nursing home care itself has been described as being “in crisis” because of the pandemic [51].

Results may look differently in regions with a higher infection rate than in the regions under investigation here. Participants were interviewed in the federal state of Mecklenburg-Western Pomerania, a rural state with the lowest infection rate in Germany, and in North-Rhine-Westphalia, a federal state with high infection rate and a higher population density. Thus, our results are more than regional results. However, samples from areas with high prevalence of Corona could be different. Further studies need to be conducted and compared to our results to get a picture of the impact and associated factors.

Our conclusions are based on cross-sectional data from a selective convenience sample from ongoing interventional trials. The strengths of this sample are, that it was accessible during the pandemic and did not have to be newly recruited. The main advantage was, that prior information on patient characteristics, cognitive and psychological status was readily available. Furthermore, the interviewed had previously – due to the ongoing trials and contact therein – established a relationship with the interviewers, which might have increased the validity of the data and might have decreased social desirability in the answers. This is important, because there can be reluctance to report attitudes and to rate services. This is illustrated by a comment of one participant: “Is [this] a check-up call to make sure we stay at home and adhere to the rules? Do you cooperate with the police? [Ist das ein Kontrollanruf, ob wir zu Hause sind und die Maßnahmen einhalten. Arbeiten Sie mit der Polizei zusammen?]”.

One needs to be careful when interpreting the association between the pandemic and our results. The data is cross-sectional and as such the pandemic cannot be interpreted as causal to changes. Only pre-post assessments could be interpreted that way. However, some of the questions asked in the interview referred to changes attributed to the pandemic. While these reports are due to hindsight-bias and retrospectively assessed, they deliver a notion of the impact. Longitudinal assessments accompanying the pandemic need to be conducted to further evaluate the impact of the pandemic on the life and health care of people.

To our best knowledge, we are not aware of a comparable sample of people with cognitive impairments living at home examined during lockdown in Germany. Thus, for comparison reasons we had to rely on related studies that had been conducted earlier. This comes with limitations in comparability. However, it gives a rough estimate how our sample differs from other samples in non-pandemic times.

In this study, we focus on a generic list of health care services and a generic list of (social) activities, which makes it difficult to compare across studies. Nonetheless, we deemed the items relevant for this particular population and chose them for inclusion in our questionnaire based on expert opinions and other studies. Interviewing elderly people with cognitive impairments by phone results in limitations. Alertness and attention over time will be more prone to deterioration than in other cohorts. Therefore, we had to choose feasibility over comprehensiveness.

The analysis relies on self-reported data. These may be biased due to the cognitive impairment. Proxy-ratings or information were obtained wherever indicated (by notion of the expert interviewer). There as no patient record available to gain a comprehensive insight of medical history of i.e. mental illnesses. This could improve the understanding of psychological symptoms occurring in lockdown. However, with the low frequency of symptoms seen in our sample, there could be methodological problems in analyzing associated factors.

## Conclusion

In summary, our study reflects the situation of people with cognitive impairment living at home during social distancing due to the Corona pandemic in Germany. It shows an only small impact of the pandemic and related measures on psychological variables like depression, anxiety and loneliness. People are well informed but not especially concerned about themselves or others. There is a decrease in social activities as expected and as intended by the restrictions imposed on the population. The impact on health care provision is significant. While this is a cross-sectional analysis, there is a need for longitudinal studies to assess mid-term and long-term effects on health-outcomes, especially for caregivers. There is also a need for comparative studies in areas with higher infection rates, in different health care systems and different countries to analyze factors associated with outcome heterogeneity.

## Supplementary Information

**Additional file 1.**

## Data Availability

Upon reasonable request, data used for this publication can be re-analyzed under the terms and conditions of the German data protection laws. Request should be addressed to the corresponding author Jochen René Thyrian.

## Abbreviations

CDC: Center for Disease Control and Prevention
DSM-IV: Diagnostic and Statistical Manual of Mental Disorders
DCM:IMPact study: Dementia Care Management: Implementation into different Care Settings-study
GAD-7: Generalized Anxiety Disorder Scale-7
intersec-CM study: Supporting elderly people with cognitive impairment during and after hospital stays with intersectoral care management
Leila75+: Leipzig Longitudianl Study of the Aged
LIFE-study: Leipzig Research Centre for Civilization Diseases
M: mean
Md: median
MMSE: mini mental status exam
PHQ-2: Patient Health Questionnaire (PHQ-2
SD: standard deviation
