# Supplementary material for: The situation of elderly with cognitive impairment living at home during lockdown in the Corona-pandemic in Germany
Source: BMC Geriatr. 2020 Dec 29;20:540. doi: 10.1186/s12877-020-01957-2 (PMC7770747; doi:10.1186/s12877-020-01957-2)
Supplement: Supplementary file 1 — Additional file 1. [file 12877_2020_1957_MOESM1_ESM.docx]

**Supplement: Items of the questionnaire used in the study**

Original language: german – translated by the authors

1. The population has been called to decrease social contacts due tot he corona pandemic. In addition, there is a contact restriction since 23^rd^ of march. Some questions in regard to this. Please answer the frequency of the following activities with more often, less often or unchanged:
   1. Being visited by family

 more often  less often  unchanged  not applicable

- 1. Being visited by neighbors

 more often  less often  unchanged  not applicable

- 1. Being visited by friend

 more often  less often  unchanged  not applicable

- 1. Meet relatives

 more often  less often  unchanged  not applicable

- 1. Treffen mit Freunde und Bekannten

 more often  less often  unchanged  not applicable

- 1. Visit birthdays/ festivities

 more often  less often  unchanged  not applicable

- 1. Visit hairdresser

 more often  less often  unchanged  not applicable

- 1. Make music

 more often  less often  unchanged  not applicable

- 1. Go dancing

 more often  less often  unchanged  not applicable

- 1. Go for coffee

 more often  less often  unchanged  not applicable

- 1. Go shopping

 more often  less often  unchanged  not applicable

- 1. Spend time outside

 more often  less often  unchanged  not applicable

- 1. Talk with friends and relatives by phone

 more often  less often  unchanged  not applicable

- 1. Watch TV

 more often  less often  unchanged  not applicable

- 1. Clean the house

 more often  less often  unchanged  not applicable

- 1. Work in the garden

 more often  less often  unchanged  not applicable

- 1. Home improvement

 more often  less often  unchanged  not applicable

1. Knitting, other crafts

 more often  less often  unchanged  not applicable

- 1. Visit the GP

 more often  less often  unchanged  not applicable

- 1. Visted by the GP

 more often  less often  unchanged  not applicable

1. Visit medical specialist

 more often  less often  unchanged  not applicable

- 1. Visit public institutions
      more often  less often  unchanged  not applicable
  2. Use computer/ tablet (Emails, Skype, Facetime)

 more often  less often  unchanged  not applicable

1. Utilize voluntary services
2. The Corona pandemic affects the health care systemand other areas of living. Please answer whether you rate your personal situation as *better, worse or unchanged in the following topics:*
   1. Mobility

 better  worse  unchanged  not applicable

- 1. Ambulatory care service Versorgung durch ambulanten Pflegedienst

 better  worse  unchanged  not applicable

- 1. Physiotherapy

 better  worse  unchanged  not applicable

- 1. Podology

 better  worse  unchanged  not applicable

- 1. Prescribed therapies

 better  worse  unchanged  not applicable

- 1. Hospital treatment

 better  worse  unchanged  not applicable

- 1. Day clinics/ day services

 better  worse  unchanged  not applicable

- 1. Provision of medication

 better  worse  unchanged  not applicable

- 1. Utilities

 better  worse  unchanged  not applicable

- 1. Food services

 better  worse  unchanged  not applicable

- 1. Relief services

 better  worse  unchanged  not applicable

PHQ 2, GAD-7,

1. When you think about the Corona pandemic Sie an die Corona-pandemie denken …
   1. Has the structure of your daily activities of living changed through Corona?

 strongly  medium  not at all  no answer

_____________________________

- 1. Are you sufficiently informed abou Corona

 yes  no  weiß nicht

- 1. Do you know anyone with Corona?

 yes  no  no answer

- 1. Do you think the measures taken to fight the pandemic are appropriate?

 yes  no, too strict,  no, not strict enough  no answer

_*welche?*____________________________

- 1. How much do you feel worried because of Corona?

 strongly  medium  not at all  no answer

- 1. How stressed do you feel de to Corona?

 strongly  medium  not at all  no answer

- 1. HHow much do you worry about your health due to Corona?

 strongly  medium  not at all  no answer

- 1. How much do you worry about the health of relatives and friends due to Corona?

 strongly  medium  not at all  no answer

- 1. How strong do you worry about Corona?

 strongly  medium  not at all  no answer

- 1. How much do you fear you could carry the infection to your relatives?

 strongly  medium  not at all  no answer

- 1. How much does it restrict you that nursing home visits are forbidden?

 strongly  medium  not at all  no answer
